# Periodic Vesicle Formation in Tectonic Fault Zones—an Ideal Scenario for Molecular Evolution

**DOI:** 10.1007/s11084-015-9411-z

**Published:** 2015-02-27

**Authors:** Christian Mayer, Ulrich Schreiber, María J. Dávila

**Affiliations:** 1 Institute of Physical Chemistry, CENIDE, University Duisburg-Essen, Universitätsstr. 5, 45117 Essen, Germany; 2 Department of Geology, University Duisburg-Essen, Universitätsstr. 5, 45117 Essen, Germany

**Keywords:** Origin of life, Supercritical carbon dioxide, Compartments, Vesicles, Molecular evolution, Tectonic fault zones

## Abstract

Tectonic fault systems in the continental crust offer huge networks of interconnected channels and cavities. Filled mainly with water and carbon dioxide (CO_2_), containing a wide variety of hydrothermal chemistry and numerous catalytic surfaces, they may offer ideal reaction conditions for prebiotic chemistry. In these systems, an accumulation zone for organic compounds will develop at a depth of approximately 1 km where CO_2_ turns sub-critical and dissolved components precipitate. At this point, periodic pressure changes caused for example by tidal influences or geyser activity may generate a cyclic process involving repeated phase transitions of carbon dioxide. In the presence of amphiphilic compounds, this will necessarily lead to the transient formation of coated water droplets in the gas phase and corresponding vesicular structures in the aqueous environment. During this process, the concentration of organic components inside the droplets and vesicles would be drastically increased, allowing for favorable reaction conditions and, in case of the vesicles generated, large trans-membrane concentration gradients. Altogether, the process of periodic formation and destruction of vesicles could offer a perfect environment for molecular evolution in small compartments and for the generation of protocells. The basic process of vesicle formation is reproduced experimentally with a lipid in a water/CO_2_ system.

## Introduction

There is no doubt that compartmentalization is a key issue in the early evolution of life, and was possibly necessary for the first living cells. In addition, it may have played a role even at a very early stage because many of the presumed steps of molecular evolution can develop much more efficiently in a confined state and compartments in principle are able to replicate (Dyson 1999; Hanczyk and Szostak 2004; Segré et al. 2001; Deamer 1997). First of all, this is valid for reactions proposed as replicator models of the RNA world (Joice 1989; Doudna and Szostak 1989; Cech 1989; Eigen 1971). In principle, some spatial compartmentalization can evolve by local diffusion processes, but this approach has been shown to have significant limitations (Nuño et al. 1995). Therefore, it is necessary to look at a scenario where, under natural conditions, small cell-like compartments can be formed in large numbers over an extended period of time.

An interesting variety of such an environment was proposed by Tuck et al. (Dobson et al. 2000; Tuck 2002; Donaldson et al. 2004): aerosol droplets could carry an exterior film of amphiphiles. When interacting with another film of amphiphiles on the surface of bulk water, a double layer can be formed leading to a vesicle structure. As a consequence, all contents of the original aerosol droplet would be encapsulated inside the bilayer membrane of the resulting vesicle. The droplets and the vesicles would have approximately the size of living cells and could be regarded as prebiotic chemical reactors. However, at the surface of the early Earth, the droplets would be subject to intense UV radiation. Therefore, sensitive organic molecules in vesicles present at the surface might deteriorate quickly. In addition, with oceans being part of the generation process of the vesicles, significant dilution of all non-amphiphilic organic components is expected, a process which would likely strongly interfere with chemical evolution.

Recently, we presented a model for the origin of life in open tectonic fault systems (mainly strike-slip faults) which offer a huge network of interconnected cracks and cavities (Schreiber et al. 2012). The sizes of the cavities range over several orders of magnitude (from sub-mm to several meters) and sum to an overall volume of several cubic kilometers. Examples for such structures are low-temperature hydrothermal systems with strong gas flux (mofettes) in the continental crust. They contain supercritical and subcritical water with various degrees of salinity as well as supercritical and subcritical gases. Here, all elements necessary for the development of prebiotic molecules may exist in varying concentrations. Furthermore, this environment combines periodically changing pressure and temperature conditions, variable pH-values, clay and transition metal-containing mineral surfaces, and a large number of other potent catalysts. Processes like the Fischer-Tropsch synthesis (which works with catalysts containing Fe, Co or Ni) or the formation of amino acids (which can be stabilized by a mixture of pyrite, pyrrhotite and magnetite) are likely under the given circumstances (Andersson and Holm 2000). While cosmic and UV-radiation are excluded due to the depth of these environments, locally occurring nuclear radiation may induce chemical reactions of the molecules inside the crust. CO_2_ which can be present as a supercritical fluid (scCO_2_) in crustal depths below 1 km (with *T* > 304 K and *P* > 74 bar) is of crucial importance. It provides a nonpolar solvent, which may be useful for example for the condensation and polymerization of hydrogen cyanide, nucleobases, nucleotides and amino acids. Inside the earliest cratons, extreme earth tides played an important role for cyclic variations within the fluid-water-interface and for the development of gradients (Cochran et al. 2004). While reactions of rising CO_2_ from volcanic sources (Lowenstern 2001) provides a constant supply of hydrothermally formed organic compounds, local pockets may be partially separated from the permanent material flow and hence could serve as long-term reaction containers. At the shallowest depths where supercritical CO_2_ occurs, a precipitation zone is expected where organic constituents may accumulate at strongly increased concentration. Altogether, the tectonic fault zone may represent an ideal environment for pre-biotic chemical evolution.

At this point, we want to introduce a mechanism of vesicle formation which is expected to occur in tectonic fault zones in presence of water and CO_2_. More specifically, it will occur at a depth where pressure and temperature conditions induce a phase transition between supercritical scCO_2_ and subcritical gaseous CO_2_ (gCO_2_) which may be around 1 km. The uncertainty derives from the fact that the density of the liquid filling of the cavities depends on the volume fraction of CO_2_ bubbles which could vary over time. With additional periodic pressure variations induced by tidal influences or geyser phenomena, a cyclic process can occur in which the transition scCO_2_ → gCO_2_ induces the formation of water droplets covered by a monolayer of amphiphilic compounds. In interaction with the interface to the aqueous domain (which by itself is covered by a layer of amphiphiles), the droplets turn into vesicles with a bilayer membrane (Dobson et al. 2000). Being thermodynamically unstable, the vesicles are expected to disintegrate and release their organic contents into the bulk water phase over time. During the transition gCO_2_ → scCO_2_, the organic constituents and the water again become soluble in the CO_2_ phase and the cycle can start again.

This leads to a continuous process of repetitive compartmentalization and remixing of water-soluble organic components. The compartments would likely have lifetimes between minutes and days and allow for extended reactions to occur under variable reaction conditions. The transient state of encapsulation is especially interesting as there are experimental indications that vesicles facilitate the formation of peptides (Tsukahara et al. 2002; Furuuchi et al. 2005) as well as their selective immobilization (Luisi et al. 1999). The described process of vesicle formation may also solve the concentration problem (which basically consists in the constant dilution of organic compounds): while an increased concentration of prebiotic molecules is already expected at the accumulation zone, the process of droplet formation leads to further accumulation of prebiotic molecules due to the low solubility of organic compounds in gCO_2_ (Williams et al. 2004).

In the following sections, we will describe the model of vesicle formation in detail and give experimental evidence for its relevance under the proposed conditions. As a model amphiphile, we have chosen a common phospholipid mixture which stands for a variety of amphiphilic compounds possibly formed under hydrothermal conditions (Rushdie and Simoneit 2006). The amphiphile is added to a two-phase system of water and CO_2_ in a high pressure apparatus where the conditions at a depth of 1 km are simulated together with periodic pressure variation. The resulting vesicles are observed and characterized by optical microscopy and pulsed field gradient nuclear magnetic resonance (PFG-NMR).

## Cyclic Formation and Dissolution of Vesicles—the Theory

The basic scenario has already been described in an earlier publication (Schreiber et al. 2012): deep-reaching open and interconnected tectonic fault systems mainly containing water and CO_2_ in the continental crust. With a wide range of pressure and temperature conditions, an abundance of catalytic surfaces and a two-phase system of water and CO_2_, it provides the perfect environment for a rich variety of hydrothermal chemistry including Fischer-Tropsch-like reactions (Dyson 1999). The constant flow of water and CO_2_ allows for the efficient transport of educts and products, while numerous isolated pockets still provide protected reaction containers. Within a depth region between 1 and 7 km, organic chemical reactions can occur in the liquid water phase as well as in the (phase separated) scCO_2_ which acts as a non-polar solvent. In addition, phase transfer chemistry is possible and allows for reactions at the phase boundary or reactions with products escaping into the adjacent phase. At the upper limit of this region (~1 km, where P and T are roughly 100 bar and 320 K), the CO_2_ turns subcritical and loses its ability to act as a solvent (the critical point of pure CO_2_ occurs at 74 bar and 304 K). At this point, all less polar or amphiphilic organic compounds will precipitate and form a variety of colloidal particles. Hereby, this relatively shallow depth region acts as a product collection zone where the concentrations of these compounds are expected to rise dramatically. Altogether, the tectonic fault system can be regarded as an efficient chemical factory with reactants deriving from hydrothermal sources in the deep crust and products collecting at the upper limit near a depth of 1 km.

**Fig. 1:**
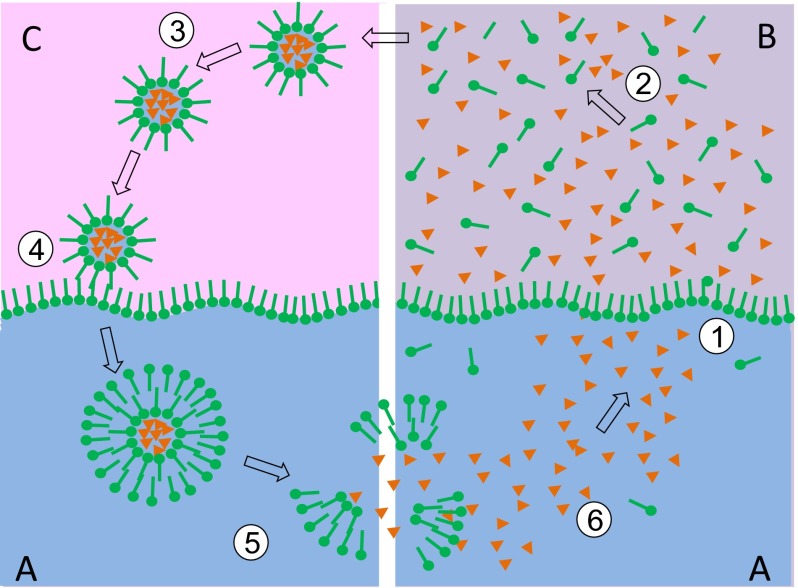
Schematic representation of a vesicle formation process in an environment formed by liquid water (*A*), scCO_2_ (*B*) and subcritical CO_2_ (*C*). The phase state of the CO_2_ changes periodically between super- (*B*, right) and subcritical (*C*, left). This induces a cyclic process including the following steps: *1*) Organic components (*orange*), amphiphilic components (*green*) and water cross the phase boundary and enter into the supercritical CO_2_ phase (*B*). *2*) The scCO_2_-phase becomes saturated with water, amphiphilic components and other organic constituents. *3*) A slight pressure drop e.g. induced by tidal activity causes a scCO_2_-gCO_2_ transition. In the subcritical phase *C*, water droplets condensate under integration of organic components and under formation of a single layer of amphiphiles. Components inside the droplets are highly concentrated and may undergo chemical reactions while the droplets of the aerosol settle onto the water surface. *4*) The single amphiphilic layer around each droplet and a single amphiphilic layer on the water surface combine to form a vesicle with a double layer membrane in the aqueous phase (*A*). *5*) The thermodynamically unstable vesicles disintegrate over time and release the organic components which have formed during step 3. *6*) The new organic materials dilute and mix in the bulk water and form the starting point for a new cycle 1-6

At this point, we focus on this upper limit region which we may further call the *accumulation zone*. Compared to the critical point of CO_2_, this region is at a slightly higher temperature (T ≈ 320 K) and hydrostatic pressure (P ≈ 100 bar). Williams et al. have shown that, at this point, a slight pressure variation can lead to an abrupt drop of the solubility characteristics of CO_2_ (Williams et al. 2004). It can further be assumed that this region is subject to periodic pressure variations, caused for example by the strong tidal phenomena of the early Earth or by local geyser activity. This given, we can assume that, at a certain depth level near 1 km, a periodic phase transition between supercritical and subcritical gaseous CO_2_ takes place. Under this influence and in presence of amphiphilic compounds, we propose that the following cyclic process will occur at the water-CO_2_ interface (steps 1 to 6 in Fig. 1):

Step 1: At this stage, the CO_2_ is in the supercritical state, so we have a two-phase system of water and scCO_2_. All organic constituents which already have accumulated in this environment now distribute between the two phases according to their individual polarity. The interface region is populated by amphiphilic components.
Step 2: The scCO_2_ phase contains less polar and amphiphilic molecules at an increased concentration. At the same time, it is saturated with water. Based on theoretical and experimental data, the water content in the CO_2_-phase can be estimated to be around 1 % under the given conditions (dos Ramos et al. 2007).
Step 3: Due to a periodic external influence like a change in tide or geyser activity, the pressure of the CO_2_-phase is slightly reduced, leading to the phase transformation scCO_2_ → gCO_2_ and hence to a drastic reduction of the solubility parameter. This causes the water to precipitate in small initial droplets with diameters of 1 μm or less. In addition, the partition coefficient for the dissolved organic constituents is readjusted in favor of the water phase. Therefore, the largest part of the less polar and amphiphilic components will be moved into the water droplets or onto their surfaces, respectively. This process is accompanied by a significant increase in concentration.
Step 4: Eventually, the droplets will settle onto the surface of the bulk water under the influence of gravity. The monolayer on their surface interacts with the monolayer on the bulk water forming a double layer with a morphology similar to a biological membrane (Dobson et al. 2000).
Step 5: The resulting vesicles disperse in the bulk aqueous phase. As the organic constituents inside the vesicles occur at a much higher concentration than in the surrounding aqueous phase, a trans-membrane concentration gradient is developed.
Step 6: Being thermodynamically unstable, the vesicles will disintegrate over time and release their contents into the bulk phase. With rising pressure (caused by the periodic influence mentioned in step 3), the CO_2_ turns supercritical again and another cycle can start with step 1.

Regarding molecular evolution, the specific advantages of this environment are manifold. They start with the significant increase of the concentration of all dissolved components. When compared to the original concentration of prebiotic molecules after their formation in hydrothermal processes at larger depths, the increase occurs in two steps: first, by the accumulation at the upper end of the depth region for supercritical CO_2_ and second, by the formation of the water droplets after the pressure decrease, leading to a new distribution equilibrium between water and CO_2_ (step 3).

Another interesting issue is the compartmentalization itself, which occurs during steps 3 to 5. Experiments have shown that condensation processes like the formation of peptides are favored by the encapsulation of the reactants in vesicles (Tsukahara et al. 2002). Further, the compartmentalization might allow for parallel molecular evolution in numerous separate containers. With an estimated number of 10^6^ droplets per cubic centimeter (derived from an average inter-droplet distance of 100 μm), this leads to a huge multiplicity of reaction environments even in a small cavity. Among these containers, the reaction conditions may differ significantly depending on the molecules which happen to be encapsulated and depending on the external conditions which may vary with the accidental location and pathway of the droplet.

With the formation of the double layer around the original water droplet, the resulting structure achieves a first step towards a cell-like morphology. The double-layer membrane exhibits a selective permeability depending on the size and the polarity of the permeating molecules (Leson et al. 2007). With the concentration gradient between the inside and the outside of the vesicle, a driving force is established which may serve as a source of free energy for all kinds of early metabolic steps.

Altogether, this cyclic process could be regarded as an ideal environment for a continuous molecular evolution. With the constant periodic change between local evolution in separated volumes and recollection of the “successful” molecules in the bulk medium over extended periods of time, with the tendency to recollect organic constituents inside small droplets and with the constant supply of prebiotic molecules from hydrothermal sources, it is hard to find an alternative environment with equally favorable conditions on the early Earth.

In the following, we report on a simple experiment which is meant to support the described model of vesicle formation. It simulates the temperature and pressure conditions at depths around 1 km in the continental crust together with a cyclic pressure variation as caused, for example, by tidal influences. The resulting vesicles were analyzed for their size and structure.

## Materials and Methods

### Materials

The materials employed were hydrogenated soybean phosphatidylcholine (NOF America Corporation, ≥98 %) as a representative phospholipid, ethanol (Fisher Scientific, ≥99.5 %) and CO_2_ (Air Liquide, 99.9995 %). The aqueous phase consisted of double-distilled water from a local source. Commercial materials were used without further purification.

High pressure experiments were performed in a custom-made phase equilibrium apparatus delivered and installed by Sitec High Pressure Technology (Switzerland). The 50 mL pressure container allows generation of pressures of up to 1000 bar with manual fine adjustment and variable temperature with stirring. Components can be added or extracted from the water phase as well as from the CO_2_ phase during experiments under constant pressure without disturbing the equilibrium conditions. The inside of the container can be inspected optically via direct observation through a sapphire window or using a camera system with an endoscope and a cold light source. A small glass insert (a cylindrical vessel with opening on the top) is used to introduce the lipid component.

### Vesicle Observation by Optical Microscopy

The aqueous phase containing phospholipid vesicles was examined by a Zeiss phase contrast microscope (Orthoplan) with a digital camera.

### PFG-NMR-Measurements of Vesicle Dispersions

The NMR experiments followed a scheme used in earlier studies on vesicle dispersions (Mayer and Bauer 2006). The ^1^H-NMR diffusion experiments were run on a Bruker DRX 500 MHz spectrometer with a Bruker DIFF30 probe head (1200 G/cm maximum pulsed field gradient). All measurements were performed at 298 K. For sample preparation, 0.4 mL of the vesicle dispersion was filled into a regular 5 mm diameter NMR tube with a 3 mm tube insert containing acetone-d6 as a source for the lock signal. As a pulse program, the stimulated echo pulse sequence combined with two gradient pulses was used. Sixteen scans were accumulated for each setting. The spacing between the two gradient pulses Δ was varied between 15 and 200 ms. The gradients were adjusted to strengths between 4 and 750 G/cm with a gradient pulse duration δ between 1.4 and 1.8 ms. All measurements (the full set of gradient strengths under variation from 4 to 750 G/cm) were repeated three times.

## Results and Discussion

### High Pressure Experiments

**Fig. 2:**
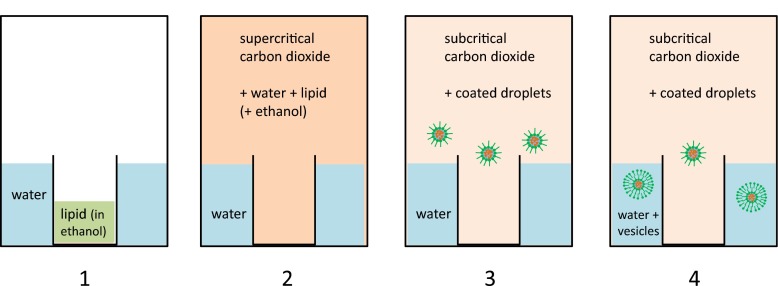
Graphic representation of the principal steps of the high pressure experiment: *1*) a high pressure vessel is partially filled with water. A small separate container positioned in the center supplies the dissolved lipid. *2*) The container is pressurized with CO_2_ until a supercritical state is reached (conditions as slightly below 1 km depth). A significant amount of water and lipid is dissolved in scCO_2_. *3*) A slight pressure drop (as in high tide situation) leads to the transformation of the scCO_2_ into the subcritical state, causing the dissolved components to precipitate as small droplets. *4*) The droplets interact with the surface of the bulk water phase to form vesicles

In an attempt to artificially reproduce the vesicle formation process described in Fig. 1, the key ingredients for the vesicles (water and the lipid) were combined with CO_2_ in a high pressure cell. Initially, the cell was filled with 5 mL of double-distilled water. In order to avoid direct vesicle formation in the aqueous phase, the phospholipid (40 mg) was dissolved in ethanol (2 mL) and introduced into the high pressure cell in a separate glass container which was positioned in the center of the pressure vessel (Fig. 2, step 1). With this setup, the cell was flooded with CO_2_ and brought to pressure and temperature conditions where a supercritical state was reached and which corresponded to the situation in the continental crust slightly below a depth of 1 km (*P* = 120 bar, T 333 K). Under these conditions, the water as well as the lipid from the ethanolic solution in the insert is expected to partially dissolve in the scCO_2_-phase (Fig. 2, step 2). Subsequently, the pressure was reduced (simulating e.g. a high tide situation) until the CO_2_ turns subcritical again (*P* = 72 bar). At this stage, the dissolved components precipitated in small droplets and the gas phase appears opaque (Fig. 2, step 3). Over time, the droplets settled onto the water surface outside the insert, saturated the interface with lipid and then interacted with this lipid layer to form vesicles. After that, the pressure was periodically cycled between 120 bar and 72 bar in order to repeatedly induce the conditions for vesicle formation. Altogether, four pressure cycles were applied.

During all these steps, all possible sources of shear were avoided in the bulk water phase. As vesicles are thermodynamically unstable, they cannot form spontaneously by diffusion of the phospholipid into the water phase. Therefore, we believe that the only possible phenomenon leading to vesicle formation in the water outside of the insert is the proposed mechanism of droplets crossing the phase boundary.

**Fig. 3:**
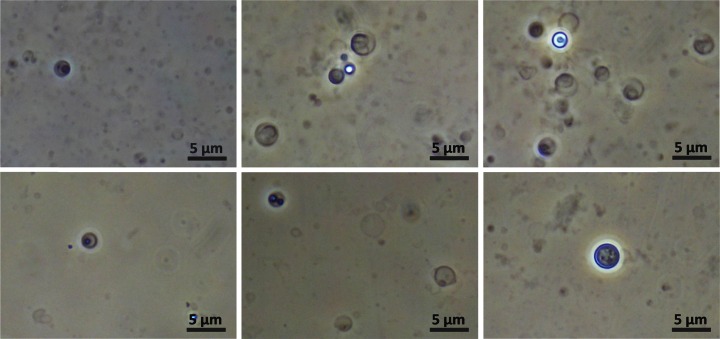
Optical micrographs of dispersed structures in the bulk water phase after the experiment described in Fig. 2

After the four cycles, the pressure was carefully released and the insert was separated. A sample of the bulk aqueous phase was studied by optical microscopy under ambient conditions. The micrograph (Fig. 3) shows spherical objects with diameters smaller than 5 μm which have the appearance of unilamellar vesicles.

In order to further identify the structure of the particles, the dispersion was studied by pulsed field gradient nuclear magnetic resonance spectroscopy (PFG-NMR). This technique allows for an estimation of the self-diffusion behavior of system components as a function of time. The intensity of the NMR echo signal I of water molecules was observed as a function of the gradient strength G and the time interval ∆ and referenced against the standard echo intensity I_0_ for *G* = 0. Commonly, the logarithm of the relative echo intensity (ln I / I_0_) is plotted vs. the parameter γ^2^δ^2^G^2^(∆-δ/3) in the so called Stejskal-Tanner-plot (with γ being the gyromagnetic ratio and δ being the duration of the gradient). In case of free diffusion of the water molecules in a bulk water system, such a plot would follow a straight line with a slope identical to the negative self-diffusion coefficient, and it would be independent on the time interval ∆. However, if water molecules are encapsulated in hollow spheres such as vesicles, they would show a completely different behavior. In this case, the slope of the plot (corresponding to the apparent diffusion constant) strongly depends on the duration of the time interval: for very short intervals ∆, it is similar to bulk water as the dislocation of the water molecules is too short to be significantly influenced by the presence of the vesicle membranes. For longer periods ∆, the average number of collisions with the membrane gradually increases, leading to a decreasing apparent diffusion constant. Finally, for very large values of ∆, the position of the water molecules averages out to the center of the vesicle and the slope of the plot represents the Brownian motion of the vesicles themselves. In this part, the intersection with the vertical axis indicates the fraction of the encapsulated molecules on a logarithmic scale.

**Fig. 4:**
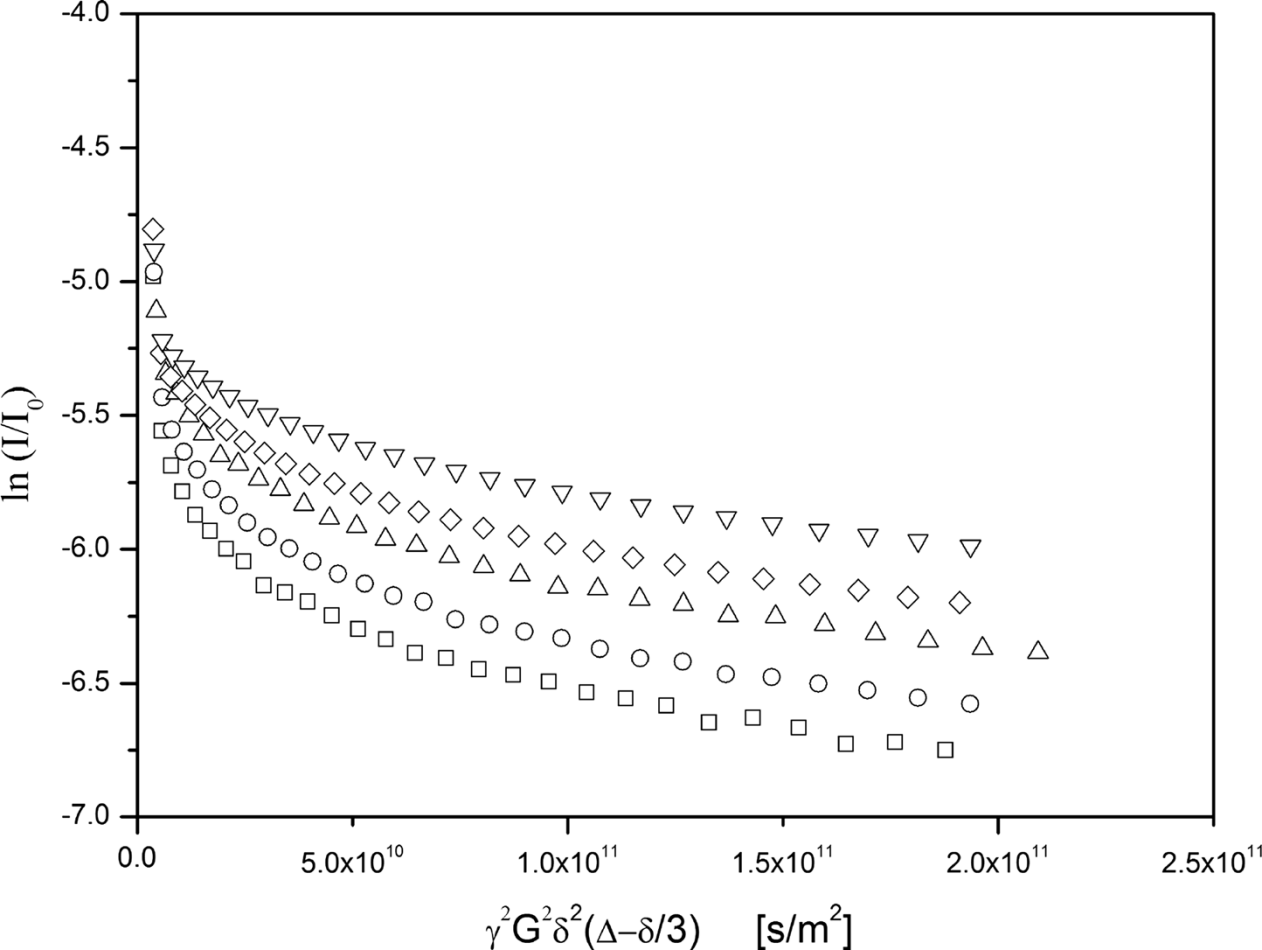
Stejskal-Tanner-plots resulting from a PFG-NMR experiment on the dispersion obtained in the pressure cell experiment. The initial drop of the relative echo intensities between 0 < ln((I/I_0_) < −5.25 is only partially shown. The five plots refer to time intervals of ∆ = 15 ms (*squares*), 25 ms (*circles*), 50 ms (*triangles*), 100 ms (*diamonds*), and 200 ms (*inverted triangles*) respectively

Figure 4 shows Stejskal-Tanner-plots obtained on the dispersion of the pressure cell experiment for different time intervals (∆ = 15 ms, 25 ms, 50 ms, 100 ms, 200 ms). All plots clearly show the presence of bulk water represented by a steep initial part of each plot, its slope indicating the self-diffusion coefficient of water (*D* = 2.3∙10^−9^ m^2^/s). However, each plot also includes a second, shallow part. Its level (and partially its residual slope) strongly depends on the time interval ∆. The initial slopes (particularly between 1∙10^10^ and 5∙10^10^ s/m^2^) clearly reflect the expected decrease of the apparent diffusion constant with increasing ∆. The terminal slopes (near and beyond 2∙10^11^ s/m^2^) are in accordance with Brownian motion based on a particle diameter of 0.5 μm. This means that the vast majority of the vesicles are smaller than the ones which could actually be observed by optical microscopy (Fig. 2). Based on the intersection with the vertical axis (which is near −5.5 for the longest ∆), the volume contribution of the vesicles can be estimated as 0.4 % of the overall bulk water phase.

Altogether, the PFG-NMR results clearly prove the presence of small dispersed compartments which are filled by liquid water. With the given system components, this leaves only one possible interpretation: the lipid has formed spherical vesicles with a bilayer structure, encapsulating a corresponding fraction of the bulk water. Vesicles are thermodynamically unstable, therefore they cannot form spontaneously. As shear was practically avoided, the only mechanism leading to vesicle formation is the interaction of lipid-coated droplets with a lipid monolayer on the water surface, as described above. Hence, we consider these data as a strong indication for the relevance of the proposed mechanism. Further, we also believe that it took place in the crust of the early Earth and presently. With the constant accumulation process linked to the initial droplet formation and the generation of a bilayer membrane, it offers a favorable scenario for early chemical evolution and the development of protocells.
